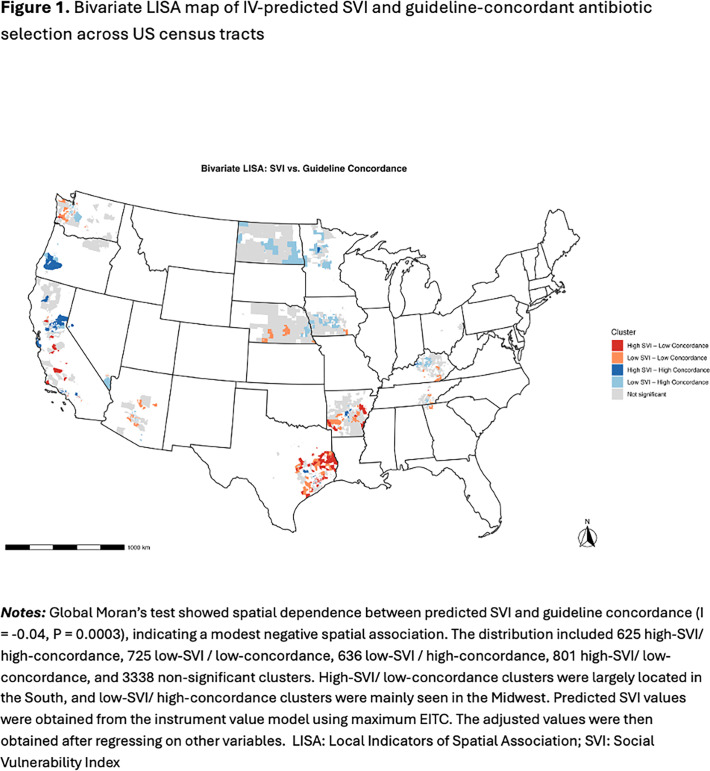# 264 Real-Time Identification and Removal of Unnecessary Vascular Access Devices to Reduce Bloodstream Infections

**DOI:** 10.1017/ash.2026.10630

**Published:** 2026-06-23

**Authors:** Mayar Al Mohajer, David Slusky, David Nix, Kasim Allel, Sabra Shay, Catia Nicodemo

**Affiliations:** 1 Baylor College of Medicine; 2 University of Kansas; 3 University of Arizona College of Pharmacy; 4 Premier Inc.; 5 University ox Oxford

## Abstract

**Background:** Older adults (≥65 years) commonly receive antibiotics in emergency departments (ED), where adherence to prescribing guidelines is inconsistent. The extent to which neighborhood disadvantage causally influences these prescribing patterns is unclear. We aimed to estimate the causal effect of community deprivation and vulnerability on guideline-concordant antibiotic selection and to disentangle the relative contributions of overuse and underuse. **Methods:** We analyzed 1,318,281 ED encounters for 790,562 adults aged ≥ 65 years seen at 119 sites across 15 states (2015–2024). The primary outcome was guideline concordance based on IDSA criteria. Secondary outcomes were overuse and underuse, defined using a three-tier diagnosis framework (tier 1 = antibiotics indicated, tier 2 = sometimes indicated, tier 3 = not indicated). Exposures were census-tract Social Vulnerability Index (SVI) and Social Deprivation Index (SDI). To mitigate confounding and reverse causality, we applied two-stage least squares instrumental-variable models using the maximum state Earned Income Tax Credit as the instrument, adjusting for patient, facility, state, and month-year covariates with patient-clustered robust standard errors. We additionally examined key SVI/SDI components and regional patterns in concordance. **Results:** Overall concordance was 83.2%; non-concordance comprised 9.5% overuse and 7.2% underuse. Concordance improved from 78.4% (2015) to 83.8% (2024), driven by declining underuse (16.2 to 5.4%) despite rising overuse (5.4 to 10.8%). Antibiotics were given in 80.8% of tier 1, 28.1% of tier 2, and 15.9% of tier 3 encounters. COVID-19 diagnoses accounted for 20.1% of overuse, and urinary-tract infection codes for 33.6% of underuse. The instrument was strong (first-stage F = 348 for SVI; 413 for SDI). In IV models, each 10-point increase in SVI corresponded to a 6.37-point lower concordance (SE 0.69; p < 0.001, Table 1); each 10-point SDI increase, 1.84-point lower (SE 0.20; p < 0.001). Associations were stronger for underuse than for overuse. Lower concordance was linked to longer travel time, higher tract non-employment, household crowding, and lack of vehicle access. Regions of high deprivation and lower concordance clustered predominantly in the South, whereas higher-concordance areas were concentrated in the Midwest (Figure 1). **Conclusions:** Community disadvantage was associated with lower guideline-concordant antibiotic prescribing for older adults, mainly through increased underuse and modest reductions in overuse. Stewardship programs should monitor both phenomena separately and target high-deprivation areas with enhanced diagnostics, follow-up, and locally tailored implementation to improve equity.

**Figure:**
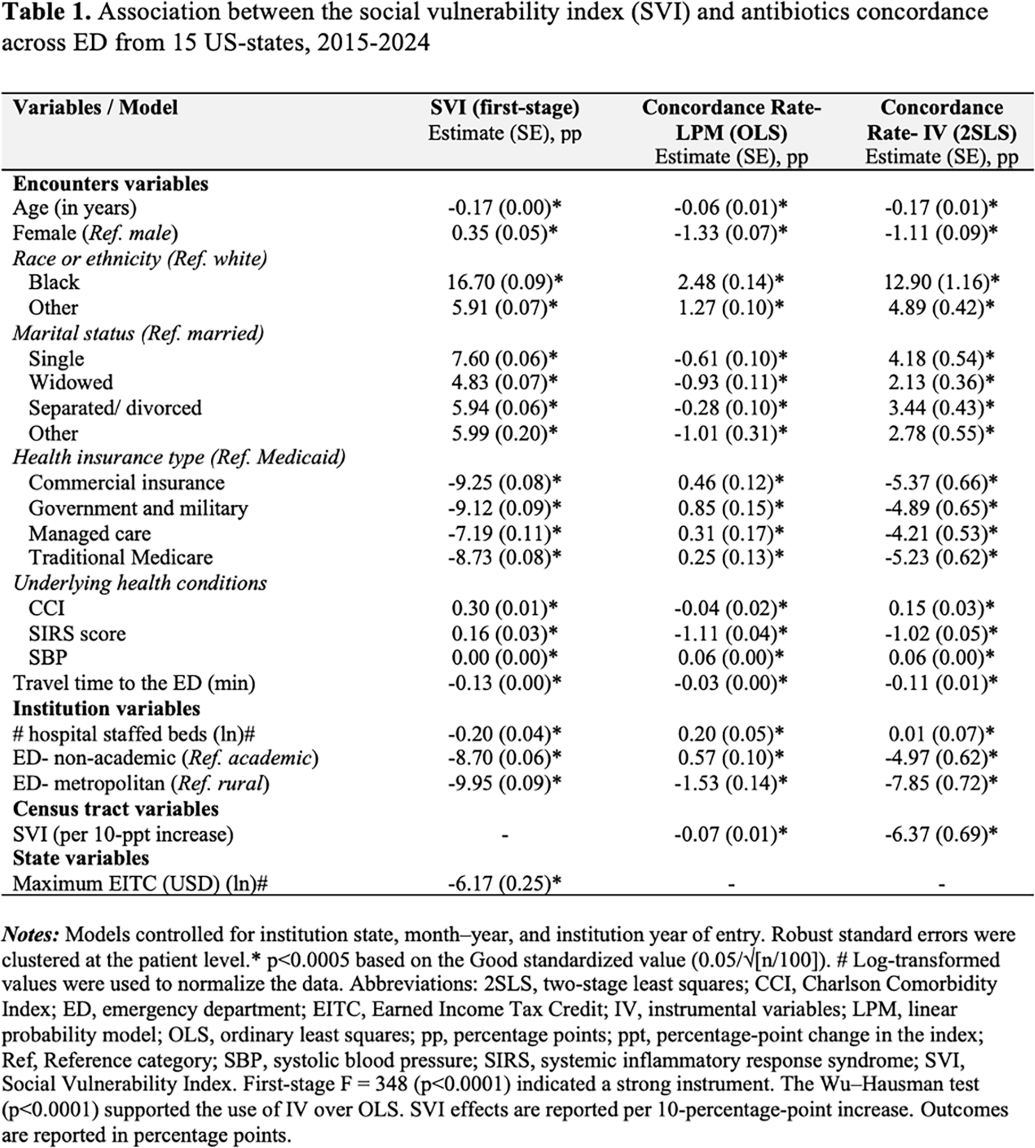

**Figure:**